# Anti-cancer & anti-metastasis properties of bioorganic-capped silver nanoparticles fabricated from *Juniperus chinensis* extract against lung cancer cells

**DOI:** 10.1186/s13568-021-01216-6

**Published:** 2021-04-26

**Authors:** Hassan Noorbazargan, Sobhan Amintehrani, Aghigh Dolatabadi, Ainaz Mashayekhi, Nazanin Khayam, Pooria Moulavi, Mohammad Naghizadeh, Amir Mirzaie, Fatmeh Mirzaei rad, Mahsa Kavousi

**Affiliations:** 1 Department of Biotechnology, School of Advanced Technologies in Medicine, Shahid Beheshti University of Medical Sciences, Tehran, Iran; 2 Department of Biology, East Tehran Branch, Islamic Azad University, Tehran, Iran; 3 Department of Biology, Tehran North Branch, Islamic Azad University, Tehran, Iran; 4 Department of Genetics, Faculty of Advanced Science and Technology, Tehran Medical Sciences, Islamic Azad University, Tehran, Iran; 5 Department of Biology, Central Tehran Branch, Islamic Azad University, Tehran, Iran; 6 Department of Biology, Parand Branch, Islamic Azad University, Parand, Iran

**Keywords:** *Juniperus chinensis*, Human lung cancer cells, Green synthesized AgNPs, Apoptosis, Metastasis

## Abstract

The current study evaluated the anti-cancer properties of bio-functionalized silver nanoparticles fabricated by *Juniperus chinensis* leaf extracts. The nanoparticles were characterized by scanning electron microscopy, transmission electron microscopy, UV–visible spectroscopy, Fourier transform infrared spectroscopy, X-ray diffraction, dynamic light scattering, Zeta potential and X-ray spectroscopy. Further, this study elucidated the cellular and molecular mechanisms of nanoparticles for anti-proliferative and apoptotic effects on human lung cancer cells (A549) and compared them with commercial drug cisplatin. The size of the spherical nanoparticle was 12.96 nm with negative zeta potential. Up-regulation of caspase 3,9 and p53, Annexin V-FITC/PI, DAPI staining, and ROS production indicated the remarkable apoptotic effect of AgNPs compared to cisplatin. Moreover, down-regulation of MMP2/MMP9 scratch and matrigel assays revealed anti-metastatic properties of AgNPs. Cell cycle analysis and downregulation of cyclin D1 indicated cancer cell cessation in the G0/G1 phase. Overall, the results revealed that the green-synthetized AgNPs had anti-metastasis and anti-proliferation effects on lung cancer cells in comparison to cisplatin with lower side effects on the normal cell line.

## Introduction

Silver nanoparticles (AgNPs) are one of the popular nanomaterials that have been progressively becoming a section of our daily lives (Singh and Sahareen 2017). Thanks to their marvelous and unparalleled nano-related features, AgNPs have been extensively applied in different fields of science especially in the biomedical field. They have also been studied for their antimicrobial effects (Iniyan et al. 2017), wound healing (Yun’an Qing et al. 2018), and anticancer (Patil and Kim 2017) activities. A variety of procurement techniques have been utilized for the synthesis of silver NPs, among which, the usage of plant extracts are more attractive due to the availability of more biological entities, eco-friendly procedures, cost-effectiveness, abundance, renewability, and safety for human therapeutic use (Kumar and Yadav 2009). A huge data evidenced that numerous phytochemicals compounds such as flavones, lignins, and terpenes can trigger anti-microbial and anticancer activities of Phyto-synthesized AgNPs (Gopinath et al. 2016).

AgNPs are utilized for treating different types of cancerous cells due to their extraordinary biomedical features (anti-cancer, anti-bacterial, anti-viral, and anti-angiogenic properties) as well as surface-specific characteristics (Burdușel et al. 2018). Various studies have indicated the apoptotic effect of silver nanoparticles on colon cancer HT29 cell line29 (Ghanbar et al. 2016), as well as the cytotoxic effects and anticancer activity of phyto-synthesized silver NPs on breast cancer MCF-7 cell line (Venugopal et al. 2017) and HeLa cervical cancer cells (Al-Sheddi et al. 2018). Further studies on mice tumors revealed that the AgNPs can significantly expand the survival time in comparison with tumor controls and consequently show satisfactory antitumor and anti-angiogenic effects (He et al. 2016). The biological results showed anti-cancerous and anti-proliferative activity of biologically synthesized silver NPs on lung cancer A549 cells. Their non-cytotoxicity can be assigned to their ability to arrest the cell cycle at the G1 phase (Annu et al. 2018). It was also confirmed that AgNPs synthesized by the *Juniperus chinensis* leaf showed the potent anticancer effect on the human adenocarcinoma gastric (AGS) cell lines (Al-Dhafri and Ching 2019).

Cisplatin is a chemotherapy drug used to treat several cancer types such as bladder cancer, head and neck cancer, esophageal cancer, lung cancer, mesothelioma, brain tumors, and neuroblastoma. Its serious side effects include numbness, trouble in walking, allergic reactions, electrolyte problems, and heart disease. This drug operates by binding to DNA and inhibiting its replication (Galluzzi et al. 2012; Zhu et al. 2016). This study was conducted to analyze the anticancer effects of green-synthesized AgNPs using *Juniperus polycarpos* on A549 (adenocarcinomic human alveolar basal epithelial cells) cell lines, as well as comparing it with Cisplatin commercial drug.

## Methods

### Plant materials and compounds extraction

The leaves of *Juniperus polycarpos* (IBRC P1010138) were obtained from the Plant Bank-Iranian Biological Resource Center. Leaves were dried under direct sunlight for 48 h and ground to make a fine powder using a stainless blender. The powder was then exposed to 500 mL of 80% methanol (MeOH) for 12 h. The extract was subsequently filtered and concentrated using a vacuum rotary evaporator at 40 °C giving rise to semi-solid extracts which were maintained at 4 °C for applying on cancer cells (Fard et al. 2018).

### Phyto-synthesis and characterization of AgNPs

Silver nitrate (0.01 M) was added to 40 ml distilled water and 4 ml plant extracts under vigorous shaking. After one day, changes in the solution color to dark brown were monitored to determine the formation of nanoparticles. The NPs were then centrifuged at 13,000 rpm for 10 min. Supernatants were discarded and the pellet was washed twice in 10 ml of distilled water to remove any contaminating plant material and centrifuged at 13,000 rpm for 15 min. At last, the mixture was dried at 37 °C for 24 h to achieve powder form.

X-ray diffraction (XRD) (PW3710, the Netherlands) with CuKα radiation (λ = 0.0260 nm) and Fourier-transform infrared (FTIR) spectroscopy were applied to analyze the crystal phase of the AgNPs powders. The AgNPs were also measured by a UV-1800UV–spectrophotometer (Shimadzu, Japan). The particle size, morphology, and distribution were detected using scanning electron microscopy (Iniyan et al.) (LEO SEM 1450VP, UK), transmission electron microscopy (Ghanbar et al.) (FEI 5022/22 Tecnai G2 20 STwin, CR), and Energy-Dispersive Spectroscopy (EDS).

### A549 cells culture

The human lung cancer A549 cell line was purchased from the Iranian Biological Resource Center. The cell lines A549 (ATCC: C137) were cultured at 37 °C under a 5% CO_2_ atmosphere. All cells tested negative for mycoplasma contamination and were markedly cultured in fresh medium (RPMI1640) supplemented with 10% fetal bovine serum (FBS, DENAzist Asia’s Co) and 1% antibiotics (penicillin/streptomycin). The cells (1 × 10^6^ cells/ml) were plated in T-25 flasks containing 5 ml RPMI1640 and grown in a humidified incubator under an atmosphere containing 95% air and 5% CO_2_ at 37 °C to the sub-confluence (90–95%) level. The culture medium was replaced every 48 h. Once the cells reached 90–95% confluency, the medium was aspirated, and the cell monolayer was washed three times with sterile phosphate buffer saline. The cell monolayer was treated with 1 ml of 0.25% (w/v) trypsin–EDTA and shortly incubated at 37 °C and microscopically visualized to ensure complete cell detachment. The cells were re-suspended in the complete growth medium. The cells were also stained with trypan blue (100 μl of cell suspension and 100 μl of 0.4% trypan blue), incubated for 2 min at room temperature, and counted using a hemacytometer. The cells were seeded at a density of 1 × 10^4^ cells/well in 96-well microtiter tissue culture plates prior to treatment with different *Vascum album* extracts (Bray et al. 2018).

### Cytotoxicity assay of AgNPs

The MTT [3-(4,5-dimethylthiazol-2-yl)-2,5-diphenyltetrazolium bromide] assay was performed for assessing cell proliferation activity and cytotoxicity in A549 cells exposed to different concentrations of bio-synthesized AgNPs and cisplatin. Cell viability was determined using the MTT assay 24 h after incubation. The MTT assay is based on the reduction of a tetrazolium component (MTT) into an insoluble formazan product due to succinate dehydrogenase activities in mitochondria. The MTT assays were conducted in 96-well plates according to standard protocols. A549 cells were seeded in 96-well plates with 1 × 10^4 ^cells/well and placed at 37 °C in a 5% CO_2_ humidified incubator until 60% confluency.

The complete growth medium was removed and the cells were exposed to serum starvation for 24 h before treatment. Cells cultures were incubated in a culture medium for 2 h alone which served as control (untreated cells) for evaluating cell viability. The cells were treated with different doses of bio-synthesized AgNPs (0.78, 1.56, 3.125, 6.25, 12.5, 25, 50 and 100 µg/ml) and cisplatin (3.125, 6.25, 12.5, 25, 50 and 100 µg/ml). Then, the medium was removed and MTT solution (100 μl) was added to each well and incubated for 4 h under 5% CO_2_ atmosphere at 37 °C for 4 h. The MTT solution was removed and 200 μl aliquots of DMSO were added to each well to dissolve the formazan crystals followed by 10 min of incubation at 37 °C. The treatments were performed in triplicates, and optical densities were read at 570 nm using a spectrophotometric method (Chen et al. 2016). IC_50_ of AgNPs and cisplatin on A549 cell lines were calculated by a statistical package (Pharm-PCS software).

### Evaluation of caspase-3, caspase-9, MMP2, and MMP9 genes expression by Real-time PCR

RNA extraction from cells was performed using an RNX kit (SinaColon Co) according to the manufacturer’s instructions. The absorbance of an RNA sample was measured at 260 and 280 nm by a spectrophotometer for calculating RNA concentration and purity. RNA quality was assessed by gel electrophoresis on a 1.2% agarose gel for 1 h at 100 V. Genomic DNA was removed by RNase-free DNase I (Thermo Scientific). The cDNA synthesis reaction was carried out with Fermentas First Strand cDNA Synthesis Kit within an RT-PCR procedure on 2 μg of the treated RNA, according to the manufacturer’s instructions. Afterward, 1 µg of cDNA was applied for the assessment of gene expression using specific primers (Table 1).

**Table 1:** Primer sequence designated for the RT-PCR

Gene	Primer sequence	Ref
Casp3	Forward: 5′- CATACTCCACAGCACCTGGTTA-3′ Revers: 5′- ACTCAAATTCTGTTGCCACCTT-3'	(Fard et al. 2018)
Casp9	Forward: 5′-CATATGATCGAGGACATCCAG-3 Revers: 5′-TTAGTTCGCAGAAACGAAGC-3’	(Fard et al. 2018)
MMP2	Forward: 5′- F: TTG ACG GTA AGG ACGGAC TC-3’ Revers: 5′-CATACTTCACACGGACCACTTG-3’	(Rahimivand et al. 2020)
MMP9	Forward: 5′- GCACGACGTCTTCCAGTACC -3′ Revers: 5′- CAGGATGTCATAGGTCACGTAGC-3'	(Rahimivand et al. 2020)
b-actin	Forward: 5′- TCCTCCTGAGCGCAAGTAC-3’ Revers: 5′- CCTGCTTGCTGATCCACATCT-3’	(Ghanbar et al. 2016)
Cyclin D1	Forward: 5′- CAGATCATCCGCAAACACGC-3’ Revers: 5′- AAGTTGTTGGGGCTCCTCAG-3’	(Choe et al. 2015)
P53	Forward: 5′- TAACAGTTCCTGCATGGGCGGC -3′ Revers: 5′- AGGACAGGCACAAACACGCACC-3'	(Lavrik et al. 2005)

The amplification was carried out in a reaction volume of 25 μL including 12.5 µl SYBR Green kit (Amplicon, Denmark), 1 µg cDNA, and 0.5 µM of each primer (Table 1) on Exicycler™ 96—Bioneer (South Korea) The temperature program was set as follows: 95 °C for 1 min; 95 °C for 15 s; and 60 °C for 60 s. β-actin gene was used as an internal control. In the current study, the 2^ΔΔCT^ method was applied to survey the relative changes in gene expression from real-time quantitative PCR experiments. Data analysis was performed with SPSS statistical analysis software and the results were analyzed by One-way Analysis of Variance (ANOVA) and Tukey’s post-hoc-test to determine significant difference between the treatments (P < 0.05).

### Apoptosis assay of AgNPs

The apoptotic toxicity toward the A549 cells was determined by Annexin- V-FITC/propidium iodide staining. According to the manufacturer’s instructions, the cells were treated with AgNPs and cisplatin for 24 h. Afterward, the cells were harvested and centrifuged at 200*g* and suspended in an appropriate buffer. subsequently, 5 µL Annexin-V-FITC labeling and 5 µL PI solutions were added to the mixture, which was then incubated for 5 min at 25 °C and analyzed with flow cytometry (Bd, UK).

### Cell cycle analysis

Cell cycle analysis was carried out on 5 × 10^5^ A549 cells (cells/mL) which were treated with *Juniperus polycarpos* leaf extract NPs and cisplatin for 30 min. Prior to staining, the cells were washed and fixed with 500 µL of ice-cold 70% ethanol and refrigerated for 1 h. The cell pellet was washed and re-suspended in 200 µL of Guava Cell Cycle reagent containing PI and incubated in the dark at room temperature for 30 min before analysis with Guava^®^ easyCyte. For each step, centrifugation was performed at 5000 rpm for 5 min at room temperature. The obtained data were analyzed using Incyte software.

### Scratch wound healing assay

Cells were seeded in a 6-well culture plate until 70–80% confluency as a monolayer, before their transfection with NPs. The scratch was vertically introduced to the cells in the monolayer using a 10-µl pipette tip. All the images were captured by OPTIKA B‐353‐PLi (Italy) microscope system at the respective time points. Each condition was assessed in triplicate and independently repeated three times.

### Matrigel invasion assay

The cell invasion assay was performed using a 24-well Transwell chamber with a pore size of 8 µm (Corning, ME). The inserts were coated with 100 µl Corning Matrigel basement membrane matrix (final concentration of 200 mg/mL, Corning, MA). Twenty-four hours after the transfection, cells (10^5^) were trypsinized, and those present in 100 µl of serum-free medium were transferred to the upper matrigel chamber and incubated for 18 h. An FBS-supplemented medium (20%) was added to the lower chamber as the chemo-attractant. After incubation, the cells passed through the filter were fixed with methanol and stained with Giemsa. The number of invaded cells was counted in 5 randomly selected high-power fields under the microscope (Li et al. 2019).

### Reactive oxygen species (H2-DCFH-DA) assay

A549 human lung epithelial adenocarcinoma cells were cultured in minimum essential medium (Hyclone Laboratories, Logan, UT, USA) containing 10 μM H2-DCFDA in a humidified incubator at 37 °C for 30 min. The cells were washed twice with warm PBS (pH 7.4) and lysed on lysis buffer (25 mM HEPES [pH 7.4], 100 mM NaCl, 1 mM EDTA, 5 mM MgCl_2_, and 0.1 mM DTT containing EDTA-free protease inhibitor cocktail (Roche)). Cells were cultured on coverslips in a 4-well plate. The cells were incubated in DMEM containing 10 μM H2-DCFDA at 37 °C for 30 min. They were again washed with PBS and mounted with VECTASHIELD Mounting Medium for fluorescence with DAPI (Burlingame, CA, USA), and imaged with a fluorescence microscope (Han et al. 2014).

### Caspases activity

The activity of protease caspases was detected using the caspase-3 (ab39401) and caspase-9 (ab65608) kits purchased from Abcam (Cambridge, UK). Caspases 3 and 9 recognize the sequence of DEVD and LEHD, respectively, and cleave from the labeled substrate p-NA emitting light, which was quantified by a spectrophotometer at 405 nm.

## Results

### Characterization of silver nanoparticles synthesized by *Juniperus polycarpos*

Changes in the color of the solution containing *Juniperus polycarpos* extract were monitored after incubation at 37 °C for 24 h. After 24 h, the solution color altered from light yellow to dark brown, suggesting the successful synthesis of silver NPs. AgNPs formation also was confirmed using UV–vis, XRD, FTIR, EDS, SEM, and TEM.

### UV–vis analysis

**Fig. 1:**
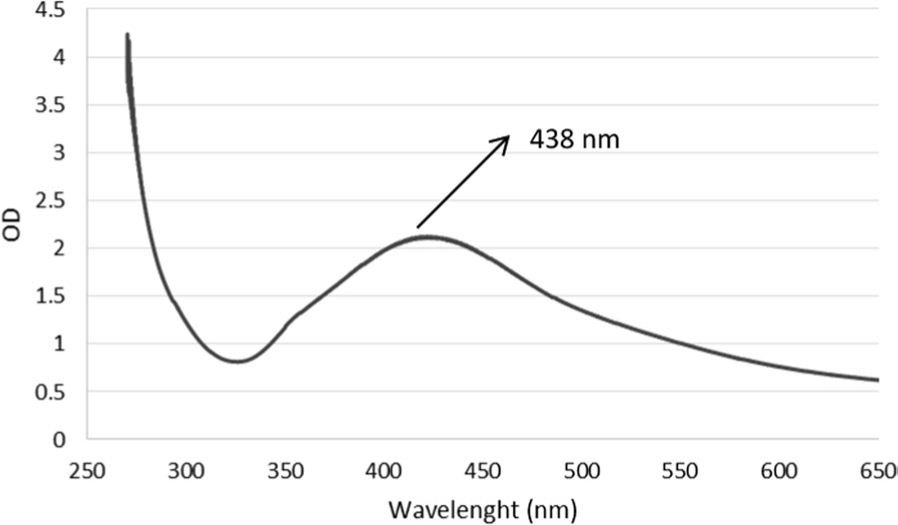
UV–visible spectrum of biologically synthesized silver nanoparticles

Figure 1 shows the UV–Vis absorption spectra of the synthesized AgNPs by *Juniperus polycarpos* extract. For UV–vis analysis, the absorption of NPs was detected at 438 nm. Moreover, no additional peaks were noticed in the spectrum, reflecting successful purification of synthesized AgNPs.

### Characterization of size and morphology of AgNPs

**Fig. 2:**
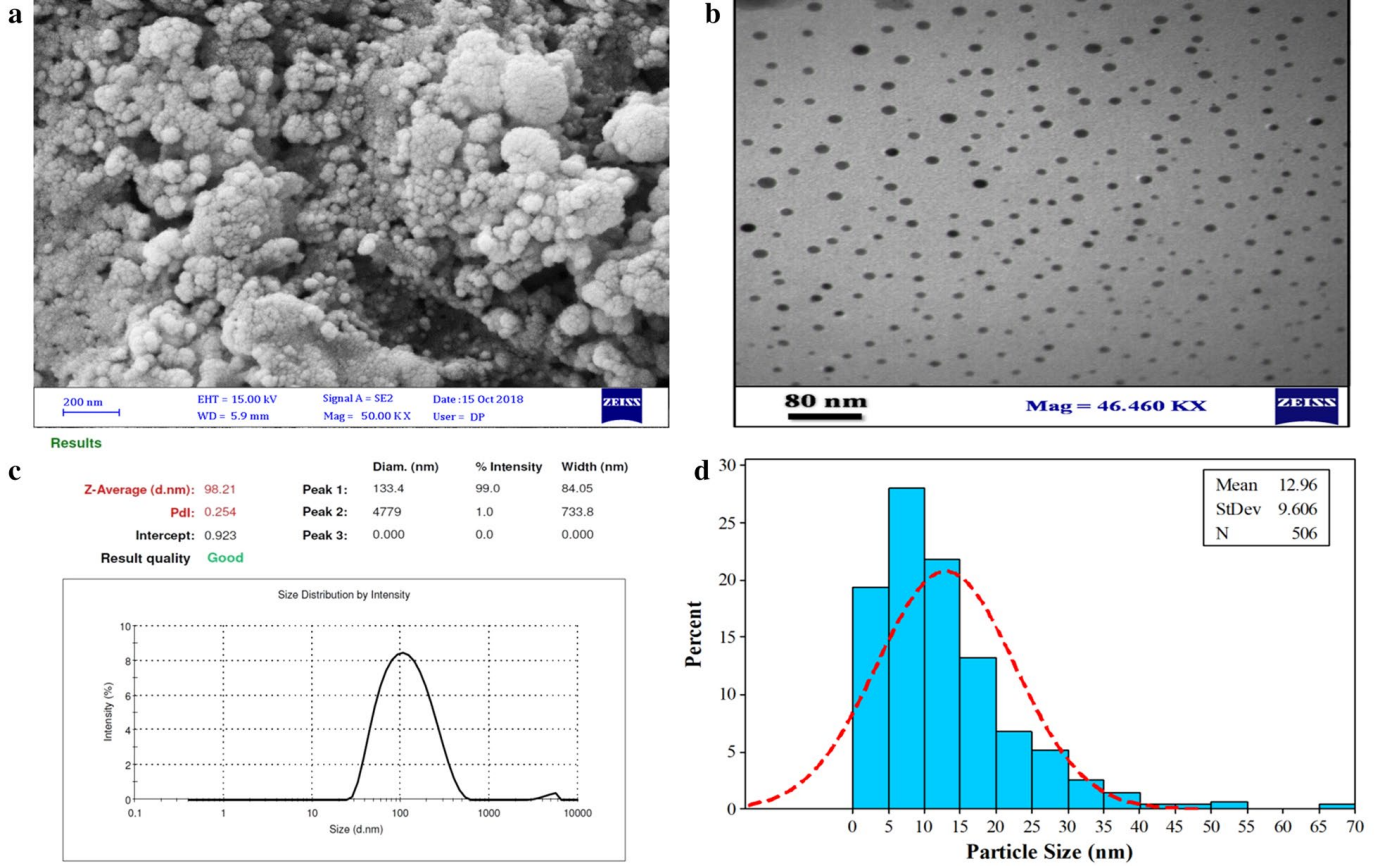
**a** SEM analysis of green-synthesized AgNPs under optimal conditions, **b** TEM micrographs of synthesized AgNPs, **c** DLS analysis, and **d** histogram of particle size

SEM and TEM images of AgNPs indicate the shape, size, and morphology of nanoparticles. According to the microscopical investigation, the AgNPs synthesized at optimal conditions had a maximum average size of 10–50 nm with a mean size of 12.96 ± 5.65 nm (Fig. 2).

According to the DLS analysis, the average size of AgNPs was 98.21 ± 1.54 nm with a polydispersity Index (PdI) of 0.254 ± 0.134.

The silver nanoparticles were found properly monodispersed with spherical shape as suggested by TEM and low PdI value of DLS analysis (Fig. 2c).

### XRD, EDX & zeta potential

The XRD patterns of the AgNPs synthesized by *Juniperus chinensis* extract indicated four intense XRD peaks at 2θ = 38.6, 44.4, 64.6, and 77.1° corresponding to (111), (200), (220), and (311) crystallographic planes of face-centered cubic (FCC) structure, respectively. The size of the synthesized nanoparticles was calculated by the Debye–Scherrer equation: D = kλ /β cosθ (Shokoofeh et al. 2019). The average size from XRD data (Debye–Scherer equation) was approximately 234@ = 23.4 nm.

**Fig. 3:**
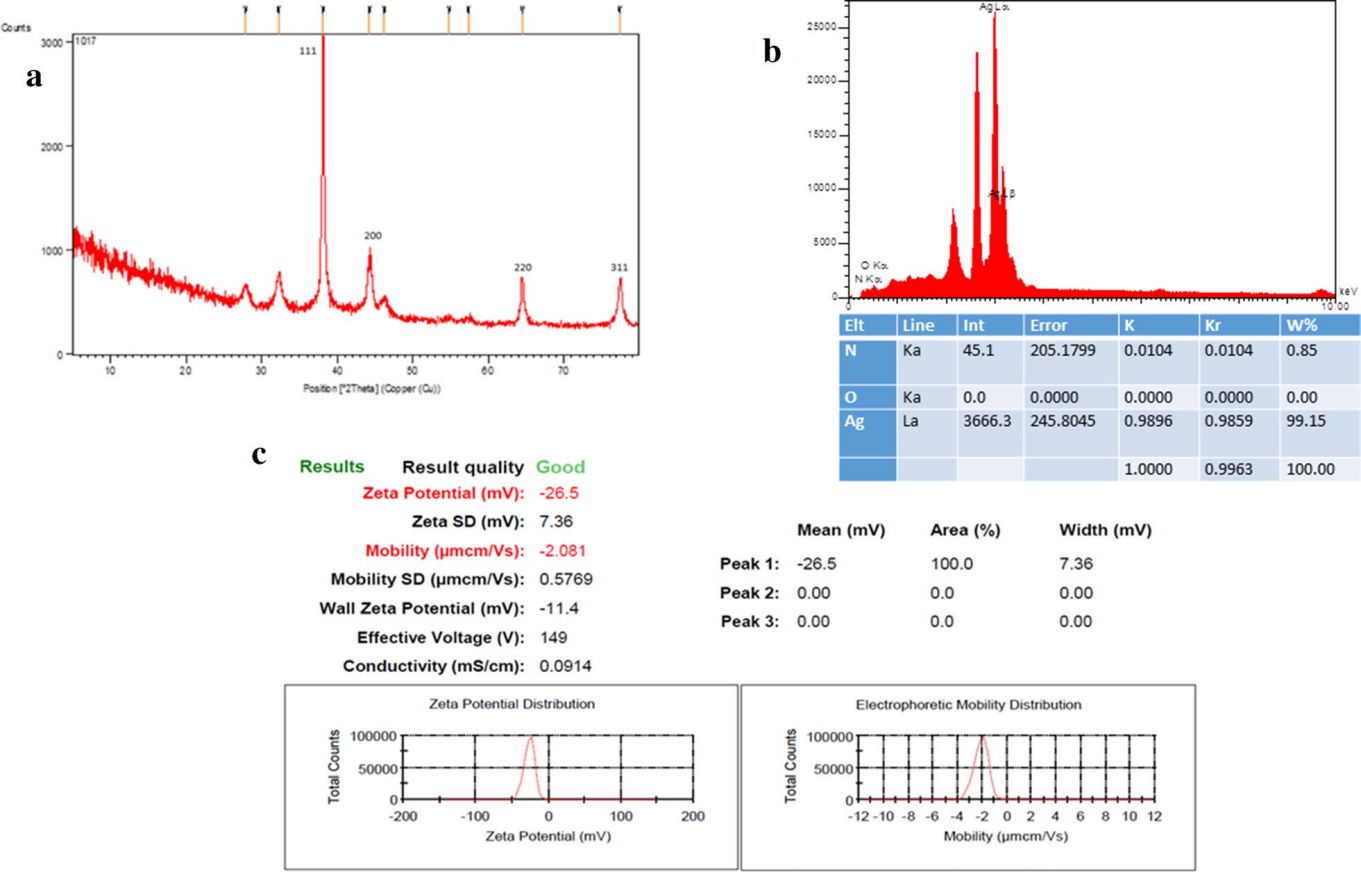
**a** XRD pattern, **b** EDS spectrum, and **c** Zeta potential of AgNPs synthesized using *Juniperus polycarpos* extract

Figure 3b shows the EDS analysis of silver nanoparticles prepared by *Juniperus polycarpos extract*. The EDS technique detects a potent signal at 3 keV indicating the presence of silver. EDS analysis also indicated the elemental analysis of the nanoparticles in which the percentage of silver ions was 99.15%.

In the current study, the fabricated AgNPs had a negative zeta potential of − 26.5 mV, indicating higher stability of the bio-functionalized AgNPs (Fig. 3c). The greater negative surface charge potential suggests that the synthesized AgNPs are well dispersed in the medium with long-term stability (Mukherjee et al. 2014).

The negative potential value may be due to the reduction and capping substances of *Juniperus chinensis* extract (e.g. fatty acid). The aggregation of the nanoparticles could be prevented by electrostatic repulsion between the negative charge of the nanoparticles (Vivek et al. 2012; Honary and Zahir 2013).

### Fourier transform infrared (FT-IR) spectroscopy

**Fig. 4:**
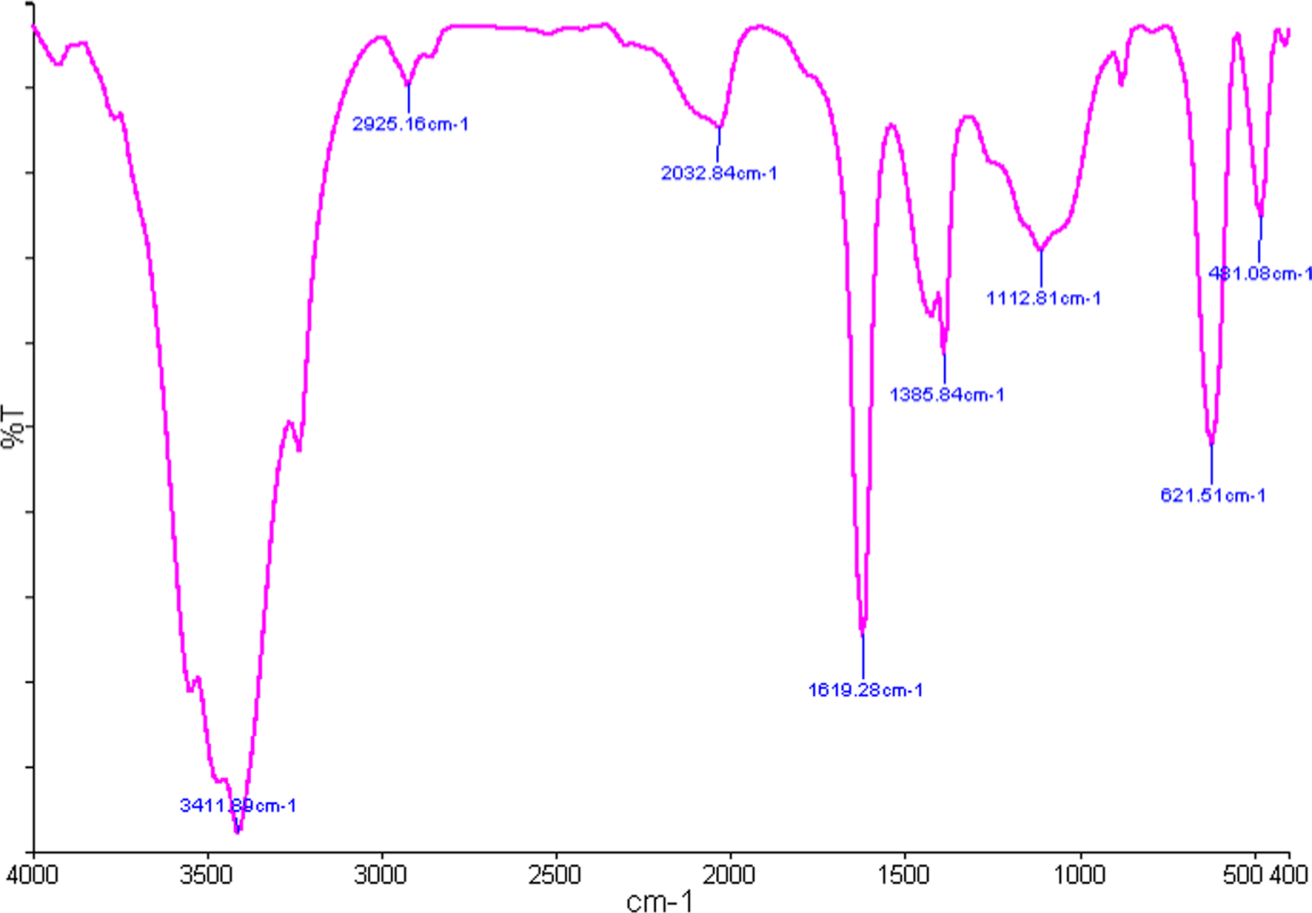
The FTIR spectrum of the AgNPs synthesized by *Juniperus polycarpos* extract

The FTIR result of bio-synthesized AgNp revealed five main functional groups: 3411.89 cm^−1^ which indicates Ar-OH H-bonding and O–H stretching; 2032.84 cm^−1^ which is representative of N = C in R-N = C = S bonds, 1619.32 cm^−1^ suggesting the out-of-plane vibration of amides RCONH_2_ NH, 1112.81 cm^−1^ which is indicative of stretching vibration of amines RNH_2_ C-N, 621.51 cm^−1^ reflecting Alkyl halide R-Br C–Br stretching. The presence of a broad and strong peak at 3411.89 cm^−1^ can be assigned to the stretching vibration of the phenolic and alcoholic O–H groups, indicating the binding of Ag to this group. A strong absorption peak at 1619.32 cm^−1^ can be also associated with stretching vibrations of the NH bond of the carbonyl amide protein group. The stretching vibrations of C–X bonds in the Alkyl halide groups appeared below 984.84 cm^−1^ and 666.10 cm^−1^. Such functional groups are involved in the capping of various plant molecules Fig. 4.

### Cell viability and MTT test

**Fig. 5:**
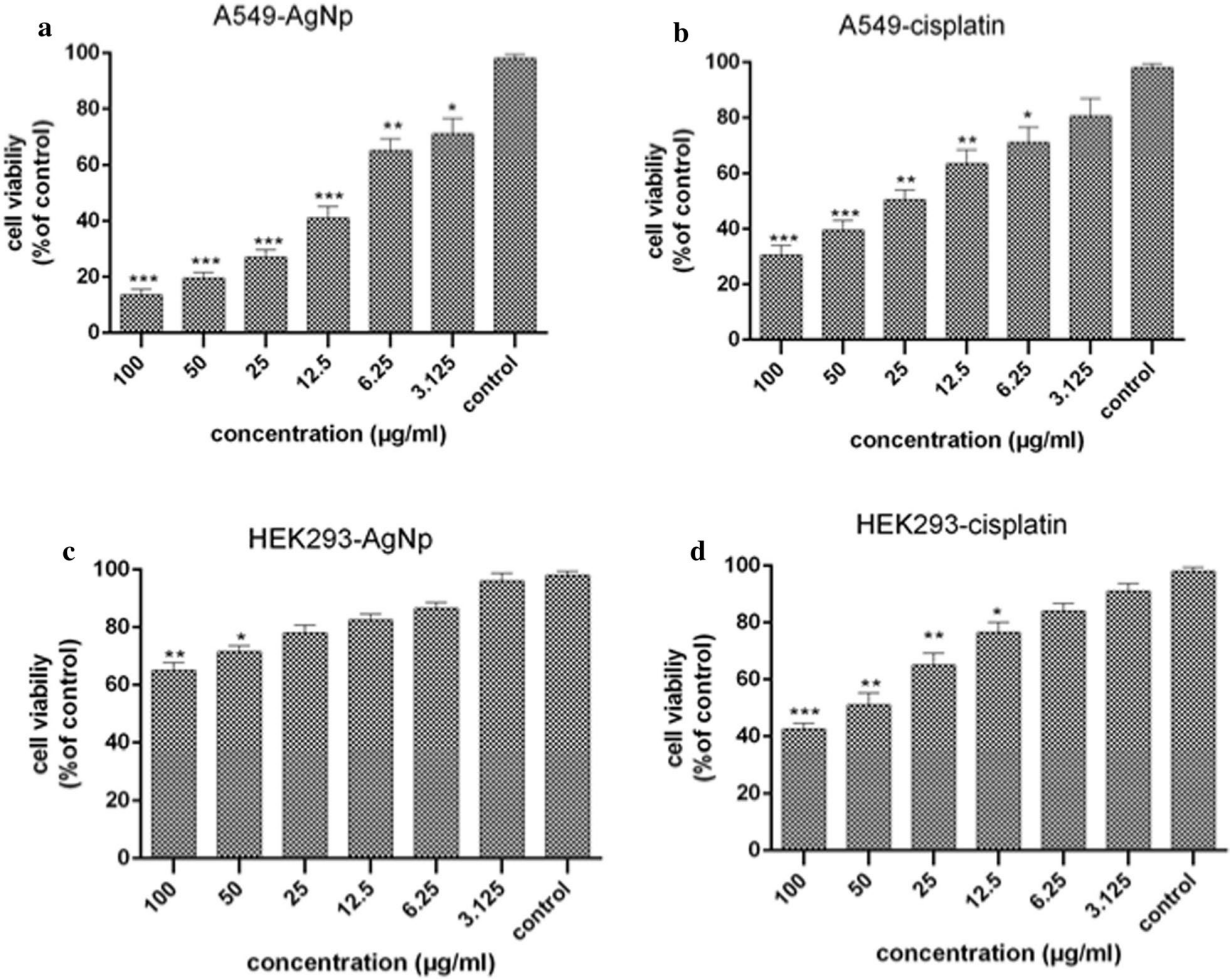
Viability of A549 and HEK293 cells upon exposure to different concentrations of bio-synthesized AgNPS and cisplatin. *indicate significant difference compared with control. (***P < 0.001, **P < 0.01 and **P < 0.05)

The possible cytotoxicity of various cisplatin concentrations and AgNPs was assessed on a normal cell line (HEK293 cells obtained from National Cell Bank (NCBI) of Pasteur Institute of Iran) and a cancerous cell line (A549). The findings indicated that AgNPs have cytotoxic effects against cancerous cell lines while exhibiting no toxicity to the normal cells at lower concentrations (Fig. 5). The maximum cell death belonged to synthesized AgNPs (35%) for the cultured HEK293 cells after 24-h exposure. Cisplatin, however, resulted in 60% cytotoxicity toward the HEK293 cells. The cytotoxicity of AgNPs and the cisplatin against MCF-7 tumor cell line were approximately 60% and 40%, respectively (at the concentration of 12.5 µg/mL). As can be seen in Fig. 5, the toxicity of AgNPs is dose-dependent.

Figures 5a, 7c indicate the viability of A549 and HEK293 cells upon exposure to 0.78, 1.56, 3.125, 6.25, 12.5, 25, 50 and 100 µg/ml of bio-synthesized AgNPs. No significant difference was observed between the viability of HEK293 cells exposed to various dosages of AgNPs. However, HEK293 cells viability remarkably decreased in the groups exposed to 50 and 100 µg/ml of AgNPs compared to control cells (P < 0.05 and P < 0.01, respectively). The reduction in the viability of A549 cells was also observed upon their exposure to 3.125 and 6.25 µg/ml of AgNPs (P < 0.05 and P < 0.01, respectively). A statistically significant difference was detected between the viability of A549 cells exposed to 12.5, 25, 50, and 100 µg/ml of biosynthesized AgNPs (P < 0.001). At the presence of silver nanoparticles, the IC_50_ values of A549 and HEK293 cell lines were calculated 9.87 and 111.26 µg/ml, respectively.

Figure 5b, d also show the viability of A549 and HEK293 cells upon exposure to various concentrations of cisplatin (3.125, 6.25, 12.5, 25, 50 and 100 µg/ml). The viability of A549 cells significantly declined upon exposure to 50 and 100 µg/ml cisplatin as compared to the controls (P < 0.001). Exposure of A549 cells to 6.25 (P < 0.05), 12.5 and 25 (P < 0.01) µg/ml cisplatin also led to a significant decrease in viability of A549 cells. However, there was no significant difference between the viability of A549 cells exposed to 3.125 µg/ml of cisplatin when compared to the controls. HEK293 Cell viability greatly decreased after exposure to 12.5 (P < 0.05), 25, 50 (P < 0.01), and 100 (P < 0.001) µg/ml of cisplatin. However, no significant differences were observed in the viability of HEK293 cells at low doses (3.125 and 6.25 µg/ml) of cisplatin. The IC_50_ values of cisplatin were determined for A549 and HEK293 cell lines as 24.67 and 43.35 µg/ml, respectively.

### Gene expression

**Fig. 6:**
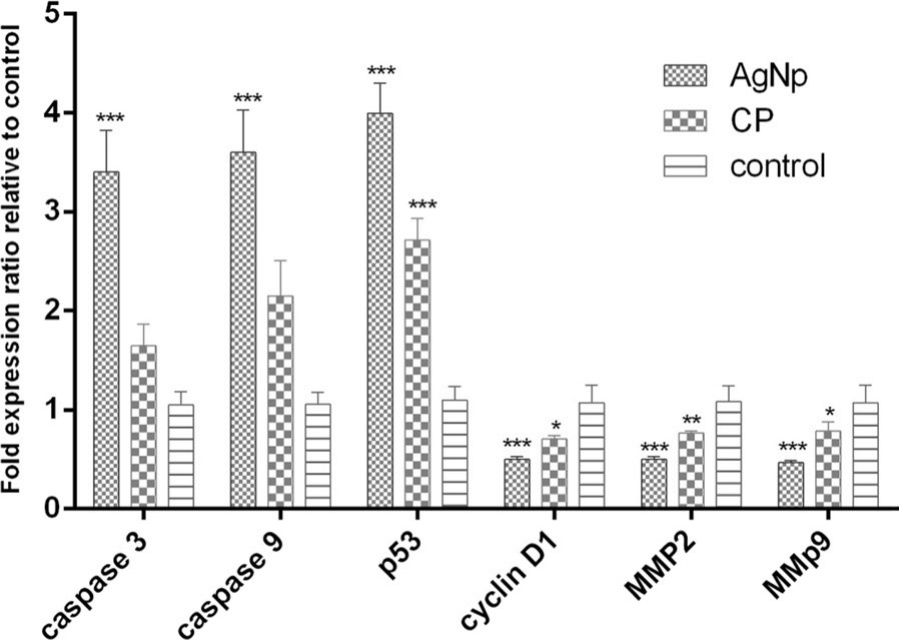
Expression levels of caspase-3, caspase-9, MMP2, MMP9, p53 and cyclin D1 genes in the cells exposed to bio-synthesize AgNPs and cisplatin. (***P < 0.001 and **P < 0.01)

Figure 6 shows the expression levels of caspase-3 and caspase-9 genes after treatment with AgNPs. According to Fig. 6, the expression levels of caspase-3 and caspase-9 were higher in cells exposed to synthetic AgNPs as compared with cisplatin-treated cells. The results also revealed that AgNPs can down-regulate the expression levels of MMP2 and MMP9 in A549 cells which were higher than the cisplatin effect. Our result also indicates the upregulation of p53 and reduction of cyclin D1 in both AgNP and cisplatin-treated A549 cells.

### Flow cytometric apoptosis analysis

**Fig. 7:**
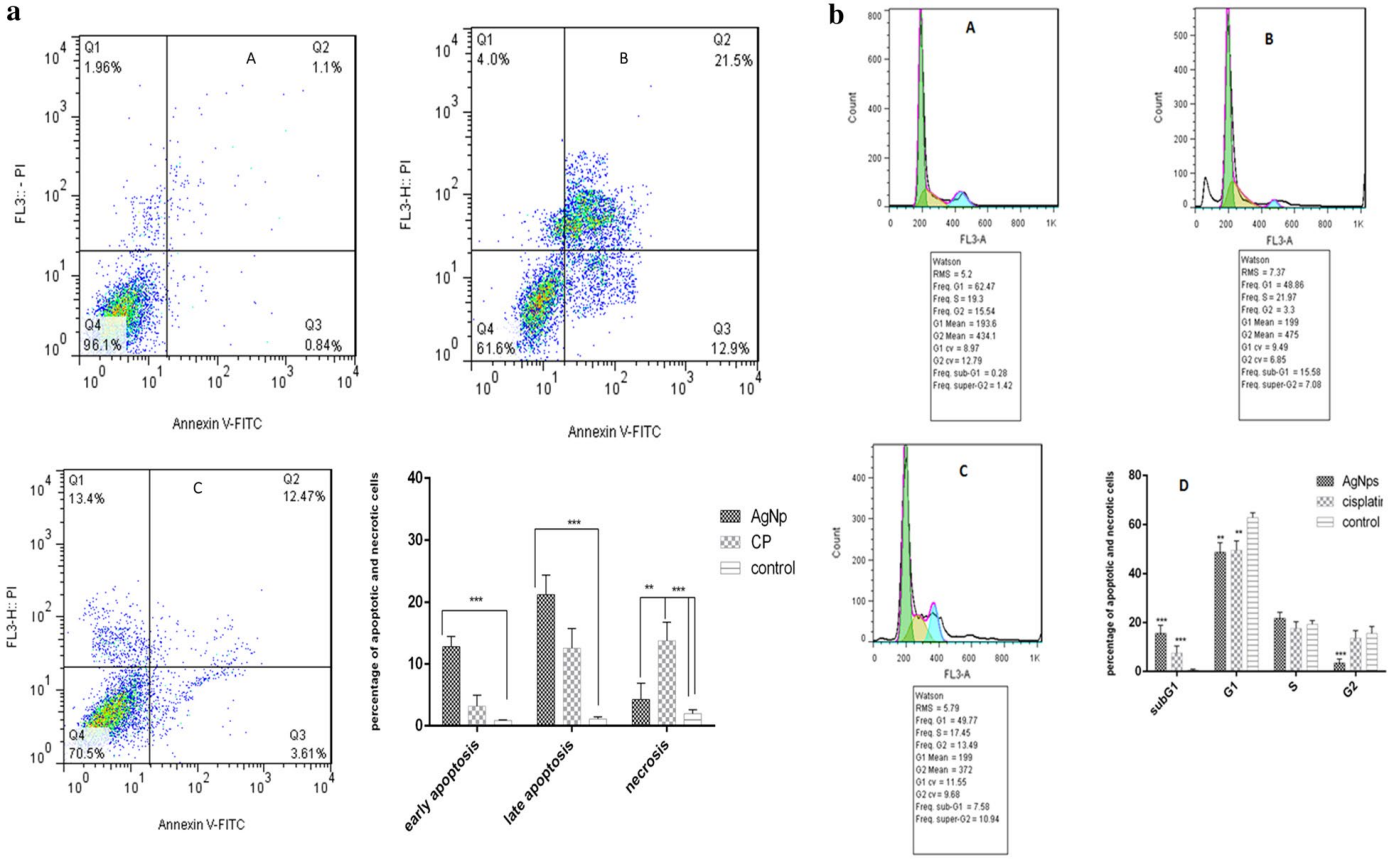
**a** Flow cytometric analysis by Annexin V-FITC in the y-axis and PI (FL3) in the x-axis double staining of A549 cell line treated with green-synthesized AgNPs and cisplatin after 24 h of exposure. Dot plots of Annexin V/PI staining are shown in (**a**) Untreated A549 cells, **b** A549 cells treated with IC_50_ concentration of AgNPs. **c** A549 cells treated with IC_50_ concentration of cisplatin

To characterize apoptosis induction of AgNPs, A549 cells were stained with Annexin-V/PI assay, followed by a flow cytometry test. The flow cytometry findings are demonstrated in Fig. 7. The flow cytometry data indicated that biosynthesized AgNPs can stimulate cell death in A549 cells. Based on the Annexin V-FITC/PI staining, 96.1% of control cells were detected viable with the early apoptotic value of 0.84%, the late apoptotic value of 1.1%, and the necrotic value of 1.9% of cells, which are common for the cells. The AgNPs-exposed A549 cells dramatically induced the late apoptotic and necrotic cells as compared with the untreated controls. An increase in the percentage of apoptotic (early and late, Q2 + Q3) cells was detected with the IC_50_ value of 34.4 and 16.08% for synthetic AgNPs and cisplatin, respectively. AgNPs had significantly (P < 0.001) lower necrotic effects (Annexin V−/PI +) (4.0%, Q1) toward A549 cells at the IC_50_ concentrations when compared with cisplatin (Annexin V−/PI +) (13.4%, Q4), suggesting of apoptotic cell death and side effects of cisplatin drug (Fig. 7a).

### Cell cycle analysis

To indicate the distribution of treated A549 cells in different phases of the cell cycle, the DNA content of cells was detected by PI staining and analyzed by flow cytometry. The results indicated that the treatment with both AgNPs and cisplatin led to an increase in the population of cells in the G0/G1 phase (Fig. 7b). The results showed that 15.58% and 7.58% of the AgNP-treated and cisplatin-treated cells were in the sub-G1 phase, respectively.

### Inhibition of migration and invasion of A549 Cells

#### Scratch wound healing assay

**Fig. 8:**
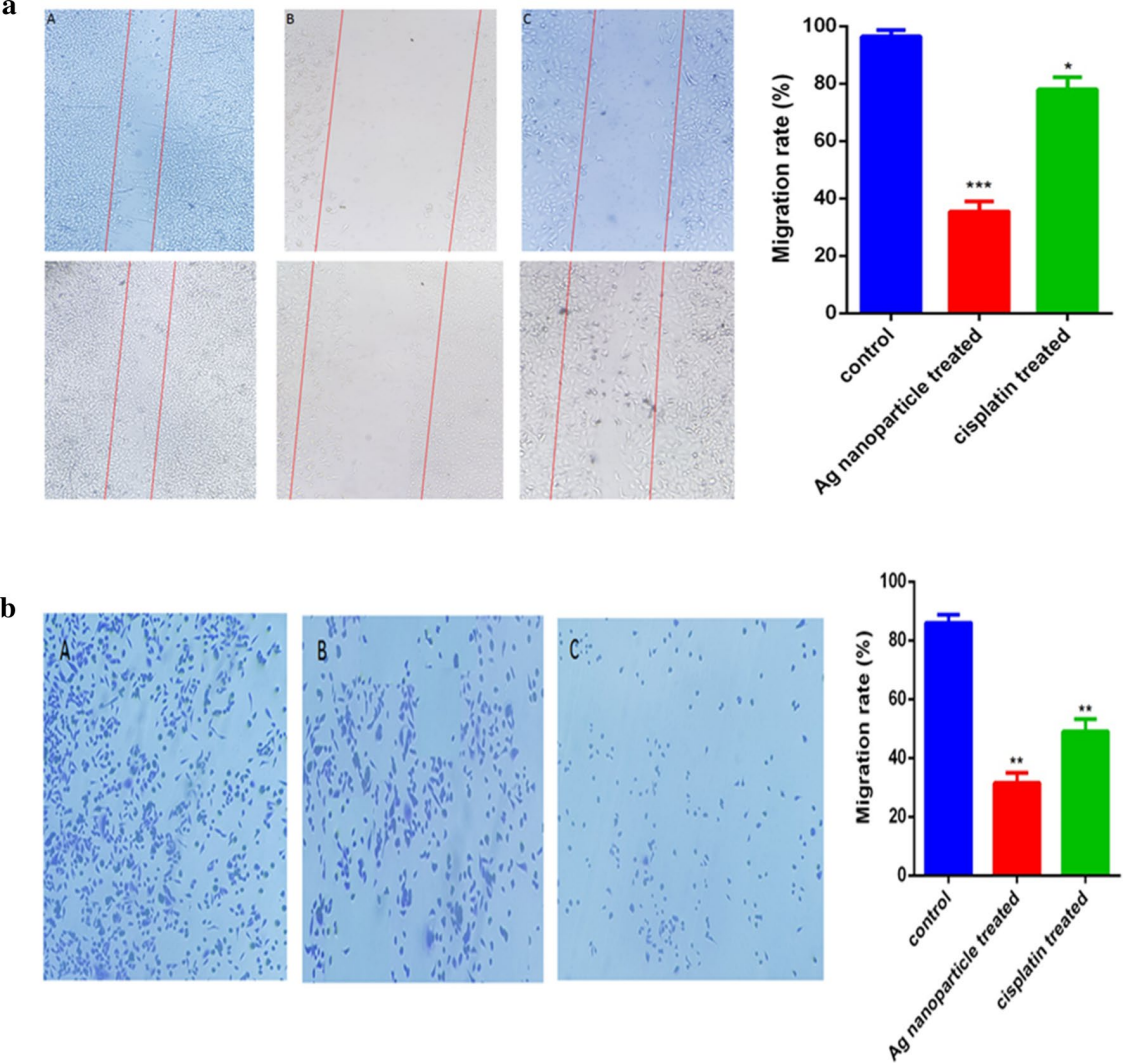
**a** Inhibition of A549 lung cancer cell lines migration in an “in vitro wound-healing” assay. **a** untreated control cells, **b** AgNPs-treated cells, and **c** cisplatin-treated cells. The bar chart shows the number of migrated cells per field The results were obtained from triplicate experiments and are presented as mean ± standard deviation (n = 3). (*P < 0.05, and ***P < 0.00). **b** AgNPs and cisplatin reduced cell mobility. the invasion rate (**a**) in control cells, (**b**) cisplatin-treated A549 cells, and (**c**) AgNPs-treated A549 cells. The results were obtained from triplicate experiments and are presented as mean ± standard deviation (n = 3). **p < 0.01

To examine the anti-metastatic properties of each drug, a scratch wound assay was performed on all three cell lines. A wound-healing assay was carried out to determine the growth inhibitory effect of AgNPs and cisplatin. Both AgNPs and cisplatin exhibited great inhibitory effects on cell migration. The wound healing results are presented in Fig. 8a. The area that the cells had migrated (toward the initially scratched midline, from the borderline) was measured. The AgNPs-treated cells migrated less than that of the cisplatin-treated and controls, indicating that both AgNPs and cisplatin weakened the migration of the A549 cells.

Figure 8 shows the migration rate in the controls and treated A549 cells. Migration ability was determined by the migration rate of migrating cells at 24 h.

#### Invasion assay

The effects of both AgNPs and cisplatin on the invasion ability of A549 cells were explored as presented in Fig. 8b. Both AgNPs and cisplatin dramatically inhibited the migration as compared with the control group. AgNPs and cisplatin decreased the migration ability from 85 to 30% and from 85 to 50%, respectively (Fig. 8b).

### Induction of caspases activation and ROS

**Fig. 9:**
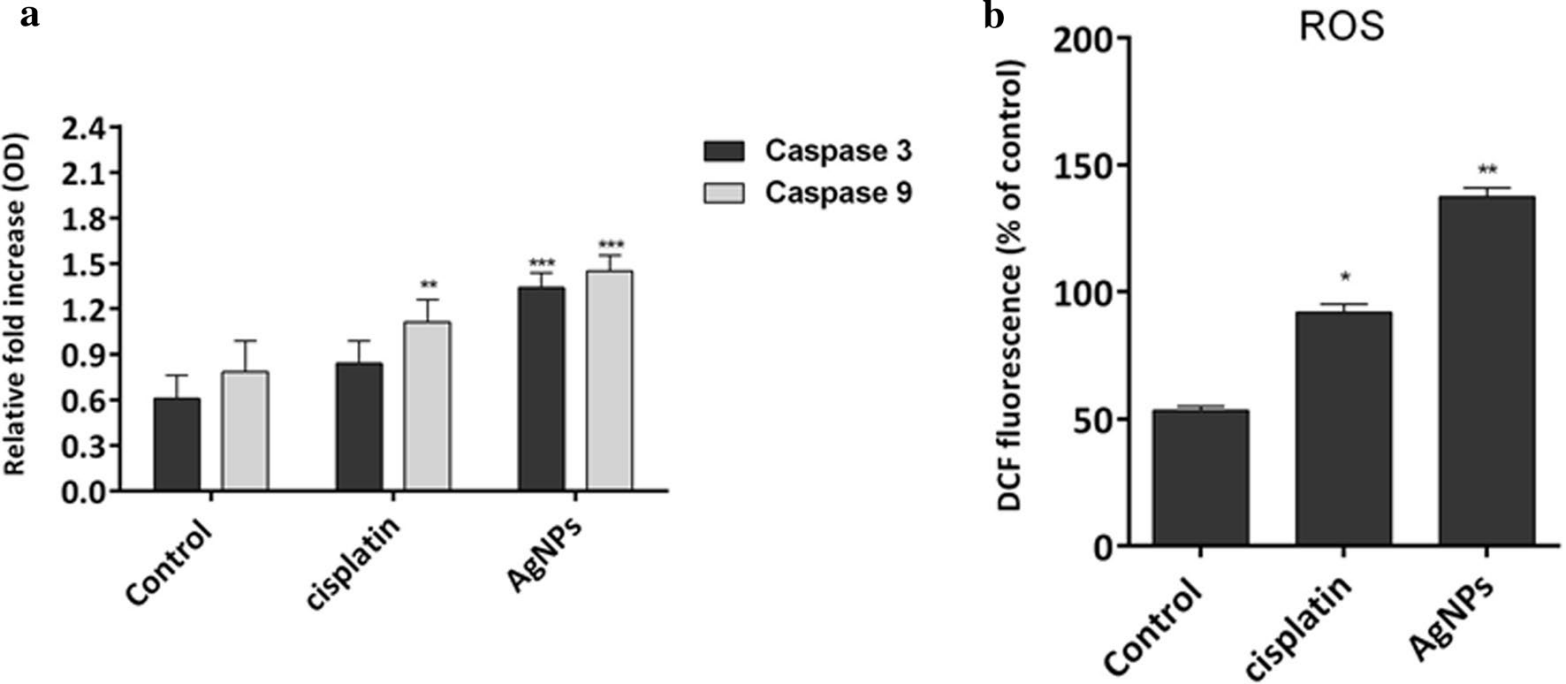
Effect of silver nanoparticles and cisplatin on caspases (**a**) and ROS production (**b**) in A549 lung carcinoma cell line. Each bar represents the mean ± SD of three independent observations. *P < 0.05 and **P < 0.01 are considered statistically significant

Caspases are the cysteine-aspartate proteases with a key role in apoptosis. Caspase-3 is the initiator caspase and caspase-9 is the executor caspase. The executor caspase expression level was increased in A549 lung cancer cell lines treated with both AgNPs and cisplatin but the caspase-3 expression level showed a significant increase only in AgNPs-treated cells (Fig. 9a). The intracellular ROS content of control, cisplatin, and AgNPs-treated A549 cells is depicted in Fig. 9b. 40% and 90% increase was observed in ROS content of cisplatin- and AgNPs-treated cells, respectively.

### Determination of apoptotic effects in A-549 cells

**Fig. 10:**
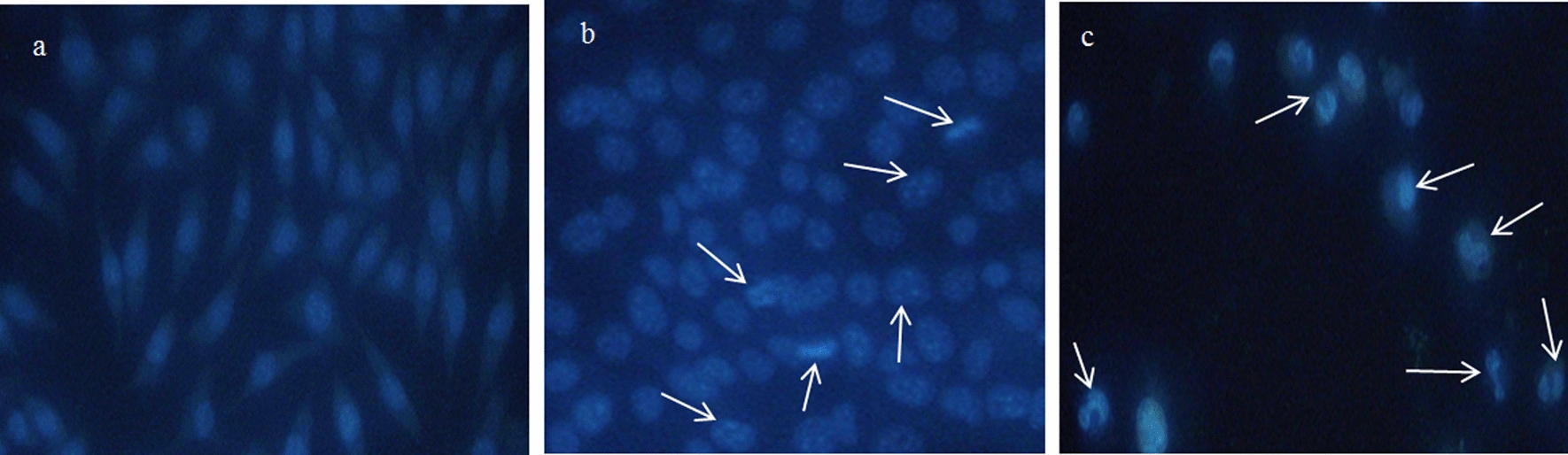
DAPI nuclear staining of (**a**) control, **b** cisplatin-treated, and **c** AgNPs-treated cells

The apoptotic pathway or programmed cell death involves several changes in the cells including their morphology, chromatin condensation, DNA, and nuclear fragmentation (Chittasupho and Athikomkulchai 2018; Rahimivand et al. 2020). To compare the effect of AgNPs and cisplatin on A549 cell death, nuclei of the cells were stained by DAPI. Figure 10 suggests higher levels of nuclear fragmentation, disintegration, and condensation of chromatin at the boundary of the nuclear membrane and cell death in AgNPs-treated cells as compared to those treated with cisplatin (Azandeh et al. 2017).

## Discussion

Cancer is a leading cause of death worldwide, accounting for an estimated 9.6 million deaths in 2018 (Bray et al. 2018). Lung cancer is the most universally occurring cancer in men and the third most common cancer in women, contributing about 11.6% of the total cancer incidence burden (Bray et al. 2018). Natural compounds possess several superiorities over synthetic drugs, including low side effects, thus, medicinal herbs have long captured the attention of researchers (Thomford et al. 2018). In the current investigation, the anticancer effects of green-synthesize AgNPs using *Juniperus polycarpos* were assayed on A549 (adenocarcinomic human alveolar basal epithelial cells) cell lines. Additionally, the expression of apoptotic and metastatic genes (caspase-3, caspase-9, MMP2, and MMP9) was evaluated.

The results showed the efficient synthesis of AgNPs using *Juniperus polycarpos* extract as confirmed by SEM, TEM, XRD, UV–vis, EDS and FTIR techniques. *Juniperus polycarpos extract* is inexpensive and easily available and can act as both reducing and capping agent in the synthesis of AgNPs. Recent studies have reported the successful use of herbal extracts for the green synthesis of AgNPs (Srikar et al. 2016). The specific mechanism of silver nanoparticles bioreduction is not fully understood yet but various investigations suggested the mechanism of metallic nanoparticle synthesis using plant extracts. Several biomolecules in the plants are engaged in the reduction and biosynthesis of metal nanoparticles (Iravani et al. 2014).

MTT assay was carried out to survey the in vitro cytotoxic trait of biosynthetic AgNPs and cisplatin. A549 cells were treated with different concentrations of AgNPs and cisplatin for 24 h to establish the inhibitory percentage against cancer cells. The findings showed that the viability of cancer and a normal cell is proportionate to the concentration of the AgNPs. Comparing the IC_50_ values of the AgNPs and cisplatin suggested the lower cell proliferation inhibition percentage of synthesized AgNPs at a lower concentration against the A549 cancer cells than the cisplatin. At lower concentrations, AgNPs demonstrated remarkable inhibitory effects on the growth of A549 cells but the cell viability did not reduce significantly at lower drug concentrations. The results of the MTT assays also showed that AgNPs exhibited lower cytotoxicity against HEK293 cells which indicated that exposure to the cisplatin is associated with increased cell death. The cisplatin–DNA intra-strand crosslinks resulted in cytotoxicity due to the presence of the platinum (Manohar and Leung 2018). Moreover, the anticancer activity of AgNPs showed a dose–response relationship and cytotoxicity increased at higher concentrations. Among the nanoparticles, AgNPs exhibited the best proliferation inhibition against cancer cells (Ghozali et al. 2015) which could be due to the synergetic properties of bio-molecular groups derived from *Juniperus polycarpos* remaining in the nanoparticles. This is the first comparative investigation to introduce the cytotoxicity of phyto-synthesized AgNPs using *Juniperus polycarpos* extract and cisplatin drug against A549 cells. Phyto-synthesis of AgNPs could ultimately lead to apoptotic cell death, DNA damage, and alterations of the cell morphology (Khalili et al 2017).

Ebrahimzadeh et al. synthesized AgNP (at the size of 25 nm) using *Anabaena flosaquae* extract, which was almost similar to the size of particles produced in this study. The IC_50_ value of AgNPs against t47d cells was 5 μg/ml and the early and delayed apoptosis were 5.21% and 34.08%, respectively. The necrosis was 28.8%, in this study, however, the IC50 concentration was 9.87 μg/ml. The early and late apoptosis were almost similar while the necrosis rate was significantly lower than the results of Ebrahimzadeh. This indicates the lower side effect of nanoparticles synthesized with *Juniperus chinensis* than Anabaena flos-aquae (Ebrahimzadeh et al. 2020).

Heidari and colleagues synthetized Ag nanoparticles with an average size of 30 nm and the zeta potential of − 12.6 mV using *Thymus vulgaris* extract. The nanoparticles in the present study were smaller and more stable (size of 12.96 nm and the zeta potential of − 26.5). Also, the apoptosis rate (34.4%) was much higher in this study as compared with the Heidari results (19.09%) (Heidari et al. 2018).

In another study, Shandiz et al. synthesized thiosemicarbazide-conjugated Ag nanoparticles functionalized with glutamic acid (Ag@Glu/TSC). The average size of nanoparticles was 50 nm and their IC_50_ was 299.1 μg/ml. The difference in the size of the nanoparticles could be attributed to the presence of the functionalized group. The IC50 was significantly low (9.87 μg/ml). The Ag@Glu/TSC induces apoptosis in 69.6% MCF-7 cells while the AgNPs of this study induced apoptosis in 34.4% A549 cells (Shandiz et al. 2018).

Considering the key role of several genes in the apoptotic and necrotic pathway, the Real-time PCR results were explored for caspase3- and caspase-9 gene expression levels in the cells exposed to IC50 of synthetic AgNPs and cisplatin. AgNPs were more powerful in decreasing the expression of MMP2 and MMP9 genes compared to the commercial drug. Various in vitro reports indicated that AgNPs decreased the mRNA and protein expression of MMP-2 and MMP-9 in wounded granulation tissues (Krishnan et al. 2018). Numerous studies have reported that treating MCF-7 cancer cells with AgNPs could lead to apoptosis by inducing the release of cytochrome c, production of reactive oxygen species (Iniyan et al.), and activation of the caspase-3 pathway (Al-Nuairi et al. 2019).

Further investigation included the annexin V/PI assay, followed by flow cytometry. In the early apoptosis stage, alterations took place at the cell surface and phosphatidylserine (PS) of the membrane is translocated from the inner to the outer leaflet of the cell membrane plasma. Annexin–V with PI staining could be used for detection of PS exposing cells via the flow-cytometry method. The groups of cells resident in the Annexin V + /PI− and the Annexin V + /PI + were identified as the early and late stages of apoptosis, respectively (Pan et al. 2014). The staining results showed that AgNPs-exposed A549 cells significantly increased the early and late apoptotic and necrotic cells as compared with untreated control cells. In contrast, the early, late apoptotic percentages of cells treated with AgNPs were found to be higher than cisplatin treatments, while the percentages of the necrotic cell (Annexin V − /PI +) was lower than the cisplatin treatment. These results indicated that AgNPs are more effective in apoptosis and have lower necrotic effects on the cells.

The anticancer properties of silver nanoparticles could be due to the wide spectrum of biological activities involved in the proliferation cycle of cancer cells, which declined the division and growth ability of cancer cells (Raja et al. 2020). The cyclin D1 and E are important in the progress of the cells from G1 to s phases (Pecorino 2016). A comparison of the cell cycle and gene expression results suggests that the treatment of A549 cells with biosynthesized AgNPs led to a considerable sub-G1 phase deterrence of cell cycle proliferation and the induction of apoptosis by downregulation of the Cyclin D1 and up-regulation of p53, caspase 3, and 9. A reduction was observed in the population of the cells treated with the AgNPs and cisplatin at the G2 and S phases, whereas the cell population in the sub- G1 phase was enhanced compared to controls. These data imply that both biosynthesized AgNPs and cisplatin progressed cell cycle development in the sub G1 phase arrest.

Regarding the contributive role of tumor cell migration and invasion in the cancer metastasis, wound healing, scratch, and invasion assays were conducted to evaluate the progression of cancer cell development in A549 cell lines, following their treatment with AgNPs and cisplatin. The results indicated that all cell lines treated with AgNPs and cisplatin invaded and migrated, but the anti-migratory effect of AgNPs in A549 cells was significantly higher than cisplatin-treated cells and control. These results proved that our targeted drug-loaded nanoparticle can be effectively used in lung cancer cell lines.

Various NPs, particularly AgNPs, can stimulate oxidative stress through ROS generation, which may induce the apoptotic pathway in response to different signals and pathophysiological conditions (Abdal Dayem et al. 2017; Nita and Grzybowski 2016). Several studies have indicated the roles of numerous metal NPs (including AgNPs) in the induction of ROS generation in many cell lines (Nita and Grzybowski 2016; Avalos et al. 2014). AgNPs have been considered as one of the most potent candidates in the medical application of nanotechnology via ROS production (El-Hussein and Hamblin 2016). This research reported a significant increase in ROS content of cisplatin and AgNPs-treated cells, indicating the initiation of apoptosis by the biosynthesized AgNP.

The current study suggested the upregulation of p53 in A549 cells upon treatment with AgNp that can arrest cells in a sub-G1 phase cell cycle and promote apoptosis induction. Downregulation of cyclin D1 stopped the cells at the G0/G1 phase and prevented their progression to the next phases. Apoptosis is evoked in arrested cells by increasing caspase 3/9 (Rahimivand et al. 2020; Fard et al. 2018; Aslany et al. 2020).

This study reported the successful synthesis of AgNPa using *Juniperus polycarpos* extract. The phyto-synthesized AgNPs showed potent cytotoxic activity against A549 cell lines as compared with cisplatin. Furthermore, the AgNPs can inhibit metastasis of A549 cancer cell lines, arrest cells at the G0/G1 phase, and stimulate apoptosis pathways rather than necrosis. Thus it can be suggested as a therapeutic agent in cancer treatment.

## Data Availability

All data generated or analysed during this study are included in this published article.
